# Treatment of abdominal pain due to deficiency syndrome of the spleen and stomach with Bian stone plus TCM iontophoresis: A case report

**DOI:** 10.1097/MD.0000000000037858

**Published:** 2024-04-26

**Authors:** Huan-Li Guo, Jing Zhao, Wen-Ying Feng, Xiao-Dong Tian, Yan-Ping Huang

**Affiliations:** a Department of Gastroenterology, Beijing Daxing District Hospital of Integrated Chinese and Western Medicine, Beijing, China; b Department of Gastroenterology, Xiyuan Hospital, Chinese Academy of Traditional Chinese Medicine, Beijing, China.

**Keywords:** abdominal pain, Bian stone plus TCM iontophoresis, deficiency syndrome of the spleen and stomach, stomachache, TCM nursing

## Abstract

**Rationale:**

Bian stone ironing and rubbing traditional Chinese medicine penetration method is based on the theory of regulating the middle and restoring balance. By using Bian stone to heat, ironing, and rubbing, pushing and rubbing in the epigastric area can regulate the spleen and stomach, restore the normal function of the middle jiao qi movement and the functions of the five organs. Bian stone hot ironing can harmonize stomach qi, nourish qi and assist yang, clear the internal organs and clear turbidity, regulate intestinal qi circulation, and promote qi stagnation.

**Patient concerns:**

The VAS score for stomach pain is 6 points, and the SAS score is moderate anxiety, which seriously affects sleep and daily life.

**Diagnoses:**

epigastric pain, spleen, and stomach deficiency cold syndrome.

**Interventions:**

Easy to digest diet, Western medicine provides famotidine acid inhibiting and protecting gastric mucosa, and mosapride promoting gastrointestinal peristalsis medication treatment; Traditional Chinese Medicine provides oral administration of Huangqi Jianzhong Tang and traditional Chinese medicine techniques such as Bianchi Ironing and Moxibustion for treatment.

**Outcomes:**

The patient’s symptoms of stomach pain have significantly improved, with a decrease in the epigastric pain score to 0, improved anxiety, reduced fatigue, improved sleep, improved epigastric fullness, unobstructed bowel movements, and improved quality of life. The patient is very satisfied.

**Lessons:**

The method of using Bian stone ironing and rubbing traditional Chinese medicine to treat stomach pain caused by the spleen and stomach deficiency cold can alleviate the symptoms of stomach pain in patients, and the improvement of symptoms shows a gradual increase, with significant effects. At the same time, it significantly improves patient anxiety and fatigue symptoms and can increase the sample size in future work to further clarify its clinical effects.

## 1. Introduction

In traditional Chinese medicine (TCM), abdominal pain is termed as epigastralgia. Individuals experiencing abdominal pain commonly present complaints encompassing symptoms such as anorexia, nausea, vomiting, regurgitation, and belching.^[1]^ According to the theory of TCM, epigastralgia is present with deficiency of stomach qi and disturbance in consuming food and digestion, and failure of the stomach qi to descend, which is caused by conditions like the pathogenic cold invading the stomach, overeating, adverse rise of liver qi, or hypofunction of the spleen and stomach. The disease can be classified into various types, including the following: deficiency and cold in the spleen and stomach, invasion of the stomach by cold, spleen-qi and stomach-qi stagnancy, and intense heat in the stomach. Among the various types, deficiency and cold in the spleen and stomach is the most common one. Abdominal discomfort of this nature has a long history, manifesting recurrently and requiring timely symptomatic treatment. Based on studies, about 80% of patients with abdominal pain have deficiency in the spleen and poor mood.^[2]^

Abdominal pain due to deficiency and cold in the spleen and stomach is caused by excessive consumption of cold food or prolonged use of medication, which causes the stomach to remain cold. The symptoms include abdominal pain, acid reflux, vomiting, loose stool, and fatigue. The treatment mainly focuses on relieving the stomach pain and warming the upper part of the body cavity to strengthen the spleen.^[3,4]^

In this case study, we report the case of a patient with abdominal pain who was treated by Bian stone plus TCM iontophoresis therapy.

## 2. Case persation

### 2.1. Clinical information

The patient, aged 72 years, with a history of recurrent epigastralgia for over 20 years was admitted to the hospital on June 12, 2023 at 09:52. She suffered from chronic gastritis, epigastralgia, and deficiency and cold in the spleen and stomach.

The patient reported experiencing recurrent epigastralgia over the course of the past decade, with a recent exacerbation of symptoms occurring within the past month following a cold. While the symptoms were manageable, they manifested 2 to 3 times daily and were alleviated through the application of hot compresses and the release of flatulence. Additionally, the patient presented with abdominal distention and nausea. The results of a physical examination at the time of admission were as follows: T 36.4 °C, HR 66 beats/min, R 18 beats/min, BP 102/56 mm Hg; the abdomen was soft without tenderness or rebound tenderness; liver and spleen were not palpable or remarkable; bowel sounds were normal. Findings from the TCM system of treatment showed that the patient was slightly over-weight and conscious with normal facial expression and lusterless complexion; the tongue was pink with a thin white coating, and the pulse was described as wiry.

According to the TCM diagnosis, the patient suffered from abdominal pain, a deficiency syndrome of the spleen and stomach.

As per western medicine, the patient was diagnosed with chronic gastritis.

The patient had no history of allergy to foods and drug.

The patient was provided secondary care, including a light diet as per the physician’s order. Famotidine and mosapride medications were prescribed to suppress gastric acid secretion and promote gastrointestinal motility, respectively. Besides, the Huangqi Jianzhong Decoction and Bian stone plus TCM iontophoresis therapy were also administered. In summary, a satisfactory outcome was obtained.

This study was conducted with approval from the Ethics Committee of Xiyuan Hospital, Chinese Academy of Traditional Chinese Medicine (2022XLA018-2). This study was conducted in accordance with the declaration of Helsinki. Written informed consent was obtained from the patient guardians.

### 2.2. Nursing

#### 2.2.1. Nursing assessment

The patient was slightly overweight and tired, she was conscious with normal facial expression and lusterless complexion; her abdomen was soft without tenderness, rebound tenderness or muscle tone; her liver and spleen were not palpable or remarkable. She complained of mild dull pain in the stomach with abdominal distention and nausea, which occurred 2–3 times daily. Her visual analogue scale (VAS) score was assessed to be 6 and her sleep was affected by abdominal pain at night.

Assessment of mental status:

The patient scored 62 on the self-rating anxiety scale, indicating moderate anxiety.

#### 2.2.2. Nursing assessment

Pain: Abdominal pain and abdominal distention.

Anxiety: Recurrent abdominal pain, poor appetite.

#### 2.2.3. Nursing plan

The treatment involved the therapy of Bian stone plus TCM iontophoresis performed 5 times per week, that is, 5 consecutive days of treatment for 4 weeks.

#### 2.2.4. Nursing objectives

The treatment aimed to ease the abdominal pain, reduce the onset frequency and the VAS score to below 3, and improve the gastroscopy result.

#### 2.2.5. Nursing measures

##### 2.2.5.1. General nursing

Environment and management of ward. The temperature in the ward was maintained between 22 °C and 26 °C and relative humidity between 50% to 60%. The patient wore loose and comfortable clothing and her abdomen was kept warm. She patient received a regular and light diet. The head of the bed was elevated at a 30° angle to ease abdominal pain and prevent regurgitation. Additionally, the patient was administered Huangqi Jianzhong Decoction orally half hour after breakfast and dinner.

##### 2.2.5.2. Nursing on emotion

① Health education. The explanatory framework delineated the involvement of deficiency and cold factors within the spleen and stomach as contributing to the manifestation of abdominal pain. Additionally, illustrative examples were presented to underscore that inadequate emotional management may exacerbate symptoms, leading to heightened abdominal pain and gastrointestinal disturbances. ② Emotion management and communication. Based on the underlying reasons for nervousness and anxiety, the patient was instructed to relax and avoid overthinking. She was also encouraged to be optimistic and confident about overcoming the disease and abandon a negative mood. ③ The following measures were recommended to ease the negative mood, if the patient felt nervous or anxious. Diverting attention: Based on neuroregulation, attention is diverted by recalling a pleasant experience or imagining a beautiful future. Hypnosis: Self-suggestion is performed by telling yourself that you can conquer the negative mood and you are the best. Confiding: If the previous 2 measures seem ineffective, the patient may resort to communication and confiding, for example, talking about pleasant topics or experience. Emotional release: When facing a negative mood, emotional release may be an effective way to relax, for example, speaking out loudly about the concerns to temporarily forget the worry.

##### 2.2.5.3. Nursing with TCM characteristics

Therapy of Bian stone plus TCM iontophoresis was provided 1 hour after breakfast.

① The temperature in the ward was maintained at 22 °C to 26 °C, with the door and window closed and the curtain drawn to protect the patient’s privacy. The patient was examined for any skin injury or skin disease. The abdomen was distended with tenderness and intestinal motility. Procedure and precautions were explained to the patient to achieve patient compliance. History of allergy was inquired. The patient was in a supine position with the abdomen exposed and kept warm.

② Three steps of therapy of Bian stone plus TCM iontophoresis.

The Bian stone ball was heated for 1 minute, and a TCM ointment formulated by blending petrolatum, paraffin oil, and powdered mixtures of TCM herbs prepared in-house by the Digestive System Department was applied on the surface of the ball and heated for 15 seconds. It was then applied on the abdomen of the patient.

Step 1: The patient’s abdomen was massaged by gently pressing down (less than 5 mm) the Bian stone ball and moving it in the clockwise direction to the dredging meridians. Keeping in mind the distention of the abdomen and the feeling of the patient, the maneuver was conducted gently for about 1 minute without causing any pain to the patient.

Step 2: The Bian stone ball was moved slowly in the clockwise direction along a spiral path based on the anatomical structure, with a slight pressure of about 1 cm. The temperature of the Bian stone ball was carefully monitored to prevent any risk of scalding the patient. After an initial 5-minute period, the therapist applied increased pressure to the Bian stone ball, pressing it deeper into the tissue, reaching a depth of approximately 2 cm to 5 cm. The therapist then gradually accelerated the movement along the spiral path for a duration of 8 minutes. Lastly, the therapist concluded the session by gently moving the Bian stone ball for an additional 2 minutes. The abdomen softened, gastrointestinal motility increased, and bowel sounds were audible. Following the release of flatulence, the patient experienced an overall sense of comfort throughout her body. The massage was performed by pressing and kneading the Zhongwan and Tianshu acupuncture points, with the maneuver applied on the Zhongwan slowly and increased gradually. The temperature of the Bian stone ball was monitored and controlled between 60 °C and 80 °C, ensuring patient comfort and to prevent scalding. Application of the temperature-controlled Bian stone ball on the surface of the skin promotes the penetration of drugs and eases muscular spasms and dredging meridians.

Step 3: The TCM cream was applied on the abdomen, covered with a plastic film and allowed to penetrate the skin for 30 minutes. The patient was instructed to relax by listening to music, and take a nap.

③ After the patient woke up, the TCM cream was removed, and the abdomen was washed with warm water. The patient felt refreshed. The therapist checked the patient’s skin and assessed the abdominal pain. There was some skin redness, which is common and normal. The patient was helped with her clothing and instructed to drink warm water to keep the abdomen warm.

#### 2.2.6. Nursing assessment

Nursing assessment was conducted based on the diagnostic and therapeutic criteria for TCM syndrome issued by the Administration of Traditional Chinese Medicine. Cure: The patient was cured of abdominal pain and other symptoms and a barium meal examination or gastroscope showed no abnormality. Response: The patient was relieved of abdominal pain, had fewer attacks and other symptoms. Barium meal examination or gastroscope showed improvement. Had the treatment been ineffective, then the barium meal examination or gastroscope would show no change. The abdominal skin of the patient was intact with reduced pain. The VAS score was assessed to be 0. The treatment yielded satisfactory results, with no abnormalities detected after 2 courses of therapy, as evidenced by improvements observed during the gastroscope examination. The patient’s sleep quality improved, and her anxiety score was normal (43) as detailed in Table 1.

**Table 1:** Comparison of symptoms.

Date of assessment	Abdominal distention	Self-rating anxiety scale (SAS)	Fatigue scale-14 (FS-14)^[5]^	Duration of sleep	Instances of stomachache	Defecation
Admission: day of admission	Significant abdominal distention; painful when pressing down 5 mm; abdomen was distended; painful during onset, score of 6	62 (moderate anxiety)	6	4 hours	2–3 times at night	Less release of flatulence, defecation every 4–5 days
After one procedure	Significant abdominal distention; painful when pressing down 2 cm; abdomen was softer than before; painful during onset, VAS score of 4	59 (moderate anxiety)	4	6 hours	2–3 times, with one instance in the night	Increased release of flatulence, defecation once
After 2 procedures	Abdominal distention; painful when pressing down 3 cm; abdomen was soft; painful during onset, VAS score of 3	54 (mild anxiety)	3	6 hours	1–2 times, with one instance in the night	Increased release of flatulence, defecation once
After one treatment course	Absence of abdominal distention; abdomen was soft; VAS score of 2	53 (mild anxiety)	3	8 hours	1–2 times, no instance at night	Increased release of flatulence, defecation every 1–2 days
After 2 treatment courses	Absence of abdominal distention; abdomen was soft; VAS score of 2	43 (normal)	2	8 hours	1 time, no instance at night	Normal release of flatulence, defecation daily

##### 2.2.6.1. Precautions

Bian stone plus TCM iontophoresis therapy should be performed between meals or 1 hour after a meal. The pressure applied during massage should be moderate and adjusted based on the softness of the abdomen and patient’s pain tolerance. The maneuver should be applied in a gradually forceful and fast manner. To promote gastrointestinal motility and metabolism, the force applied on the Bian stone ball should be gradually increased with the patient’s abdomen getting soft and the pain decreasing.

The therapy is conducted for 20 minutes. The patient may feel tired if the therapy continues for a longer time; if the duration is too short, the efficacy may not be obtained. During the treatment, the patient’s comfort is considered. While initially, the abdomen is distended, it becomes soft after releasing flatulence and the patient feels relaxed. The therapist adjusts the maneuver and force applied according to the patient’s comfort and ensures that the temperature of the ball is optimal to prevent scalding of the skin. The patient is instructed not to worry about the skin redness since it is normal. The patient is encouraged to ambulate and relax by listening to music.

### 2.3. Treatment outcomes

Following 2 courses of treatment involving Bian Stone therapy coupled with TCM iontophoresis, a noteworthy reduction in abdominal pain symptoms was observed. The VAS score demonstrated a substantial decrease, reaching 0, indicative of a complete alleviation of pain. Moreover, anxiety and fatigue levels were diminished, accompanied by enhanced sleep quality and the absence of abdominal pain, leading to regular bowel movements. The patient reported satisfaction with an overall improvement in life quality.

## 3. Discussion

Systematic and safety therapies for abdominal pain have been developed during the evolution of TCM in the past centuries.^[6,7]^ According to the theory of TCM, abdominal pain can be divided into various types: pathogenic cold retained in the stomach, exhaustion of stomach yin, and deficiency and cold in the spleen and stomach. In clinical practice, the prevailing form of abdominal pain arising from deficiency and cold in the spleen and stomach constitutes the most prevalent category. In accordance with historical medical counsel, practitioners traditionally addressed this type of abdominal pain through the administration of Huangqi Jianzhong Decoction.

In TCM, abdominal pain is referred to as epigastralgia. The stomach is considered the organ responsible for receiving food and initiating digestion, and it is closely interconnected with the spleen in terms of its exterior and interior relationships. Both constitute the postnatal foundation. In case of improper diet, moodiness, or invasion of pathogenic cold, the failure of stomach qi to descend may occur with abdominal pain. As stated in the chapter Wu Xie of Ling Shu, an ancient Chinese medical text, “when the evil energy is in the spleen, the muscle of the patient will be painful.” Ancient physicians tended to advocate the principle of warming the middle-energizer to strengthen the spleen, and the principle of regulating qi and stomach.

The 15 minute therapy of Bian stone plus TCM iontophoresis consists of the following steps: 1. Applying the TCM cream on the surface of body, massaging with the Bian stone so that the maneuver and the drug exert a comprehensive effect to achieve the treatment of disease. Temperature-controlled Bian stone ball when applied on the surface of the skin serves to promote the penetration of drugs, easing muscular spasms, and dredging meridians. The heat of the Bian ball helps to promote gastrointestinal motility and ease abdominal distention. 2. Cream application: Drugs in the TCM cream not only increase the transdermal effect of the drug, but also play a lubricating role. Through various physical effects such as its medicinal properties and temperature, it can improve abdominal qi and blood circulation, play a role in temperature meridian circulation, drive away cold and pain, and improve satisfaction.^[5,8,9]^

Pressing and kneading the Zhongwan and Tianshu acupuncture points can invigorate the spleen, harmonize the stomach and promote the middle-energizer. The term Tianshu implies a continuous stream. As stated in the “Compendium of Acupuncture and Moxibustion,” “Tianshu is also termed as Changxi and Gumen, which is located 1 inch away from the Huangshu, and is positioned 2 inches apart on both sides of Qi Zhong. It is the Front Mu point of the large intestine.” Tianshu is the acupuncture point of the Stomach Meridian of Foot-Yangming. The stomach and spleen are interconnected in terms of their exterior and interior relationships, and together, they form the basis for postnatal functioning in TCM. Pressing and kneading the acupuncture point can effectively regulate the transportation and transformation of the spleen and promote the release of flatulence. Zhong Wan is an acupuncture point of the Conception Vessel and the Front Mu point of stomach, as well as the meeting point of the abdomen, which is responsible for harmonizing the stomach and regulating the function of the middle-energizer. Massaging the abdomen: This action helps in clearing any obstructions in the flow of blood and qi and promotes the movement of healthy qi to combat pathogenic factors.^[10]^

The “Huangqi Jianzhong Decoction” was first recorded in the “Synopsis of Golden Chamber,” a monograph on the diagnosis and treatment of miscellaneous diseases in China. The formulation consists of milkvetch root, malt sugar, cassia twig, debark peony root, Chinese date, Glycyrrhizae Radix Et Rhizoma Praeparata Cum Melle, and Zingiberis Rhizoma Recens. The collective action of the 7 drugs operates synergistically to invigorate the middle-energizer and reinstate the balance of qi. This therapeutic formulation is specifically recommended for the alleviation of abdominal pain associated with deficiency and cold in the spleen and stomach.^[8]^ Huangqi Jianzhong Decoction is effective in the treatment of abdominal pain by both promoting circulation of qi and blood to ease the pain and reinforcing qi to invigorate the spleen and harmonize the stomach.

The combination therapy of Bian stone massage and TCM iontophoresis proved to be effective in gradually and significantly alleviating the symptoms of the patient experiencing abdominal pain attributed to spleen and stomach deficiency with a cold nature. Furthermore, this treatment approach was found to have a positive impact on the patient’s anxiety and fatigue levels. However, it is important to acknowledge the limitations of this study. This study only described 1 case, which may affect the generalizability of the research results. Potential constraints or challenges accounted for during the research process, such as sample selection bias or data collection limits, should be addressed. To ensure the robustness and reliability of the results, future studies should consider conducting trials with larger sample sizes to validate the effectiveness of this therapy more comprehensively. Given the strong policy support for promoting TCM nursing practices in China, healthcare professionals have the opportunity to actively contribute to the advancement of TCM nursing by leveraging TCM resources.^[11]^

## Acknowledgments

We would like to acknowledge the hard and dedicated work of all the staff that implemented the intervention and evaluation components of the study.

## Author contributions

**Conceptualization:** Huan-Li Guo, Yan-Ping Huang.

**Data curation:** Huan-Li Guo, Jing Zhao, Xiao-Dong Tian.

**Formal analysis:** Jing Zhao, Wen-Ying Feng, Xiao-Dong Tian, Yan-Ping Huang.

**Writing – original draft:** Huan-Li Guo.

**Writing – review & editing:** Jing Zhao, Yan-Ping Huang.
